# Prognostic Significance of Serum Interleukin-6 Levels in Oral Squamous Cell Carcinoma

**DOI:** 10.7759/cureus.54439

**Published:** 2024-02-19

**Authors:** Tomoko Adachi, Hiroyuki Goda, Satoru Shinriki, Norihiko Tokuzen, Nobuyuki Kuribayashi, Satoshi Hino, Koh-ichi Nakashiro, Daisuke Uchida

**Affiliations:** 1 Department of Oral and Maxillofacial Surgery, Ehime University Graduate School of Medicine, Ehime, JPN; 2 Department of Molecular Laboratory Medicine, Faculty of Life Sciences, Kumamoto University, Kumamoto, JPN

**Keywords:** prognostic markers, nutrition status, disease free survival (dfs), overall survival (os), oral cavity squamous cell carcinoma, interleukin -6

## Abstract

Introduction

The prognosis of oral squamous cell carcinoma (OSCC) is often poor despite standard treatments. Additionally, no useful prognostic markers are available. Therefore, we aimed to investigate the relationship between serum Interleukin-6 (IL-6) levels and prognosis and explore its local and systemic effects in patients with OSCC.

Methods

Ninety-five new cases of OSCC were included, and the prognosis was compared between high and low serum IL-6 groups. The localization of IL-6 in OSCC tissues was examined. Furthermore, a comprehensive gene expression analysis was performed in OSCC tissues and compared between the two groups.

Results

A significant difference in overall survival and disease-free survival was observed. Furthermore, a substantial expression of IL-6 was localized in the stroma. Comprehensive gene expression analysis of tumor localization showed increased expression of genes related to oxidoreductase and lipid metabolism in the primary tissues of the group with high serum IL-6 levels. Regarding the correlation between blood tests and serum IL-6 levels, a strong positive correlation was observed between inflammatory responses and nutritional factors.

Conclusion

These results suggest that serum IL-6 may be a prognostic factor for metabolic abnormalities in patients with OSCC and that aggressive nutritional interventions may contribute to prognosis.

## Introduction

Oral squamous cell carcinoma (OSCC) is one of the most common malignancies with an estimated yearly incidence of over 400,000 new cases worldwide [1]. Despite advancements in technology and therapies that enhance patients’ quality of life, the overall prognosis for patients remains unsatisfactory due to the frequent occurrences of local recurrences and distant metastases. Despite significant efforts, the survival rate has remained unchanged for the past three decades [2]. The prognosis of OSCC patients is influenced by various factors, including primary tumor size, lymph node metastasis, distant metastasis, and histopathological grade [3]. The prognostic significance of multiple biomarkers in OSCC has been the subject of numerous studies, yet the results have been inconsistent [4]. Therefore, the identification of biomarkers that can predict prognosis is being sought.

We previously reported the predictive utility of serum Interleukin-6 (IL-6) levels as a predictor of prognosis in patients with early-stage OSCC defined by sentinel node biopsy [5]. IL-6 is a versatile cytokine predominantly produced by T cells, macrophages, fibroblasts, and monocytes. It has a crucial role in multiple biological processes, including inflammation, immunity, hematopoiesis, differentiation of nervous system cells, and proliferation of renal mesangial cells [6]. The IL-6 receptor forms a complex with glycoprotein 130 (gp130) upon binding to IL-6, resulting in the dimerization of the gp130 molecule. This triggers the phosphorylation of Janus Kinase (JAK) associated with gp130, leading to phosphorylation and dimerization of signal transducer and activator of transcription 3 (STAT3). The dimerized STAT3 then translocates into the nucleus and regulates the transcription of target genes. IL-6 is also implicated in acquiring malignant features in various cancers, including OSCC [7]. The relationship between high levels of IL-6 and poor prognosis in OSCC has been reported previously [8]. However, the underlying mechanism of this association and its clinical significance are poorly understood. This study aims to evaluate the predictive value of serum IL-6 levels in OSCC.

## Materials and methods

Patients

This study was carried out at the Ehime University Hospital and included 95 cases of freshly diagnosed patients with OSCC who were diagnosed between December 2014 and December 2021. All patients underwent surgical treatment, and none had received prior radiation or chemotherapy. The study received ethical clearance from the institutional review board (no.1308006 and no.1309015), and written informed consent was obtained from all participants. Participants who were excluded from the study were those with a history of other malignancy, distant metastasis at the time of diagnosis, and inability to obtain a blood sample.

Serum collection and IL‑6 enzyme-linked immunosorbent assay (ELISA)

Serum was collected before surgery and frozen at -80°C until used for the IL-6 assay. According to the manufacturer’s protocol, serum IL-6 levels were analyzed using a human IL-6 ELISA kit (R&D Systems, Inc. Minneapolis, MN, USA). A cut-off value of less than 8 pg/mL was used as the standard value for the assay.

Cells and cell culture

This study used three human oral cancer cell lines: SAS, HSC2, and HSC3. These cells were grown in Dulbecco's modified Eagle's medium (DMEM; Wako, Osaka, Japan) supplemented with 10% fetal bovine serum (FBS; Biosource, Camarillo, CA, USA), 100 U/mL penicillin and 100 μg/mL streptomycin (Wako), referred to here as the complete medium. The cells were cultured at 37°C in an atmosphere containing 5% CO2, and when the cell density reached approximately 60%, they were washed twice with PBS, the medium was changed to serum-free, and after 48 hours, the supernatant was collected and cell counting was performed simultaneously.

Fibroblast cell lines were established from a tissue sample obtained from an 84-year-old Japanese male diagnosed with well-differentiated OSCC. Written informed consent was obtained, and tissues were retrieved from two distinct regions: the OSCC tissue and non-malignant oral mucosa tissue. After surgical excision, the tissue was rinsed several times with phosphate-buffered saline (PBS) and cut into small fragments. The pieces were then cultivated in a complete medium and incubated at 37°C in air containing 5% CO2. The technical procedure was performed as described previously [9].

Immunohistochemical staining

Immunostaining was conducted using antibodies targeting vimentin, α-SMA, and cytokeratin AE1/AE3 (Dako, Glostrup, Denmark) to verify the identity of the isolated cells as fibroblasts. Cells were fixed with 10% formalin for 10 minutes and then blocked with 3% bovine serum albumin (BSA)/PBS. Primary antibodies against vimentin (1:80), α-SMA (1:100), and cytokeratin AE1/AE3 (1:80) were applied for 60 minutes at 37°C. HRP-conjugated secondary antibody (Dako Envision System; Dako) was then applied for 60 minutes at 37°C. Finally, the cells were stained with a DAB substrate and counterstained with hematoxylin.

Immunohistochemical analysis was performed on surgically resected OSCC specimens using the avidin-biotin-peroxidase complex method. Formalin-fixed samples were embedded in paraffin, cut into 4μm sections, and deparaffinized in xylene before being rehydrated in descending concentrations of alcohol. Endogenous peroxidase activity was blocked by incubating the sections with 0.3% hydrogen peroxide for 15 minutes. The sections were washed three times with phosphate-buffered saline and incubated overnight at 4°C with a specific polyclonal antibody against human IL-6 (diluted 1:3000; ROCKLAND, Gilbertsville, PA, USA). Subsequently, the sections were overlaid with the biotinylated anti-rabbit antibodies at room temperature for 30 minutes, washed in phosphate-buffered saline, and labeled with streptavidin-peroxidase complex. The peroxidase reaction was developed using 3,3-diaminobenzidine as a chromogen. The sections were counterstained with hematoxylin, dehydrated with ethanol, treated with xylene, and enclosed in synthetic resin.

Semiquantitative analysis of the immunohistochemically stained sections was performed in a blind fashion by evaluating three randomly chosen fields in each sample. The samples were scored based on the immunoreactivity scoring system (IRS) [10], which calculates the product of the percentage of positive cells (4, >80%; 3, 51-80%; 2, 10-50%; 1, <10%; 0, 0%) and the intensity of the staining (3, strong; 2, moderate; 1, mild; and 0, no staining) resulting in IRS scores between 0 (no staining) and 7 (maximum staining). The cancer cells and stromal cells stained with anti-IL-6 antibodies in the cytoplasm were counted. The evaluation was conducted based on the average of the assessments made independently by two oral surgeons.

Samples from patients

For microarray analysis, tumor tissue specimens were obtained from patients during surgery. Total RNA was extracted using ISOGEN (NipponGene, Tokyo, Japan) by the manufacturer's protocols, utilizing TissueLyser (Qiagen, Valencia, CA, USA). The integrity of the extracted RNA was verified using Agilent 2100 Bioanalyzer (Agilent Technologies, Santa Clara, CA, USA).

Microarray analysis

The conversion of total RNA to digoxigenin (DIG)-labeled cRNA was accomplished using the Applied Biosystems Chemiluminescent RTIVT Labeling Kit (Life Technologies, Carlsbad, CA, USA). cDNA was generated using 1 μg of total RNA, transcribed using DIG-labeled nucleotides (Roche Diagnostics, Basel, Switzerland), fragmented, and hybridized to Human Genome Survey Arrays (Life Technologies) as per the manufacturer's protocol. After washing each array, the signal was developed using a chemiluminescent detection kit (Life Technologies). The processed arrays were scanned on a 1700 chemiluminescent microarray analyzer (Life Technologies), and the results were analyzed using DAVID analysis wizard (https://david.ncifcrf.gov/tools.jsp, Frederick, MD, USA). The raw microarray data have been deposited in Gene Expression Omnibus (GEO, http:// www.ncbi.nlm.nih.-gov/geo, experiment nos. GSE254525) according to the minimum information about microarray experiment (MIAME) guidelines.

Inflammatory reaction and nutrition assessment of OSCC patients

Before the initiation of treatment, blood samples were collected to measure various biomarkers such as C-reactive protein (CRP), albumin, white blood cell count, and neutrophil, lymphocyte, and platelet (Plt) count levels. An Inflammatory reaction and nutrition assessment was calculated for each patient using a combination of several indicators such as the Glasgow Prognostic Score (GPS), Neutrophil/Lymphocyte Ratio (NLR), Plt/Lymphocyte Ratio (PLR), Prognostic Nutritional Index (PNI), and CRP/Albumin Ratio (CAR).

Statistical analysis

Statistical analysis was carried out using GraphPad Prism statistical software version 5 (GraphPad Software, Inc., La Jolla, CA, USA). The continuous variables were presented as the mean ± standard deviation. The Chi-square and Student's t-test were used to compare categorical and continuous variables, respectively. The Kaplan-Meier method was utilized to calculate overall survival (OS) and disease-free survival (DFS), which were then compared using the log-rank test. Multivariate Cox proportional hazards regression analysis was performed to identify independent prognostic factors. A P-value of less than 0.05 was considered statistically significant.

## Results

Characteristics of the patients

This study determined the established threshold for IL-6 serum levels was 8 pg/mL, based on the general reference values. Among the 95 patients diagnosed with OSCC, 17 patients had elevated serum IL-6 levels, whereas 78 patients had low IL-6 levels. As shown in Table 1, the median age of the participants was 69.8 years, with 50 (52.7%) being male and 45 (47.3%) being female. The primary sites affected were mainly the tongue and mandibular gingiva, followed by maxillary gingiva, buccal mucosa, and oral floor. There were no significant differences in serum IL-6 levels according to the primary site of the tumor. Although no correlation with serum IL-6 levels was observed for differentiation degree and mode of invasion, a significant correlation was observed for pStage and T classification.

**Table 1:** The Relationship between patient characteristics and serum IL-6 levels

Number of patients		Serum IL-6 high n=17	Serum IL-6 low n=78	P-value
Age	median (range)	69.8 (29-88)		
	Male	7 (7.4%)	43 (45.3%)	0.7284
	Female	10 (10.5%)	35 (36.8%)	
T classification	1	2	17	0.0279 *
	2	4	39	
	3	5	7	
	4	6	15	
N classification	－	11	60	0.3571
	＋	6	18	
M classification	－	17	77	>0.9999
	＋	0	1	
pStage	Ⅰ	2	18	0.0278 *
	Ⅱ	2	30	
	Ⅲ	5	8	
	Ⅳ	8	22	
Y-K	1	1	1	0.4920
	2	1	14	
	3	10	46	
	4C	5	16	
	4D	0	1	
Grade	1	7	44	0.2554
	2	9	25	
	3	1	9	
Primary site	Tongue	3	29	0.5370
	mandibular gingiva	7	21	
	maxillary gingiva	3	13	
	buccal mucosa	2	6	
	floor of the oral cavity	0	5	
	Mandible	1	2	
	Lip	1	1	
	Palate	0	1	

Kaplan‑Meier survival plots comparing serum IL-6 levels and the clinical outcome

**Figure 1:**
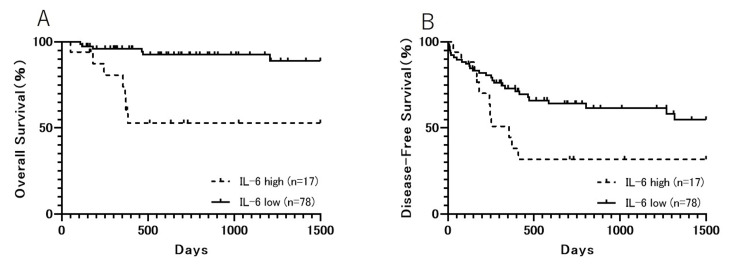
The survival rates of patients with OSCC. (A) Overall survival (OS) in the interleukin-6 (IL-6) high and low groups. The median OS in the IL-6 low group was significantly higher than that in the IL-6 high group (P<0.0001). (B) Disease-free survival (DFS) in the IL-6 high and low groups. The median OS in the IL-6 low group was significantly higher than that in the IL-6 high group (P<0.0283).

A group comparison of serum IL-6 levels for OS and DFS was performed using the Kaplan-Meier method. Our findings indicate that a higher serum IL-6 concentration is associated with a significantly lower OS (P<0.0001) and DFS (P=0.0283) in patients with OSCC (Figure 1A and B).

Localization of IL-6 in OSCC tissue

**Figure 2:**
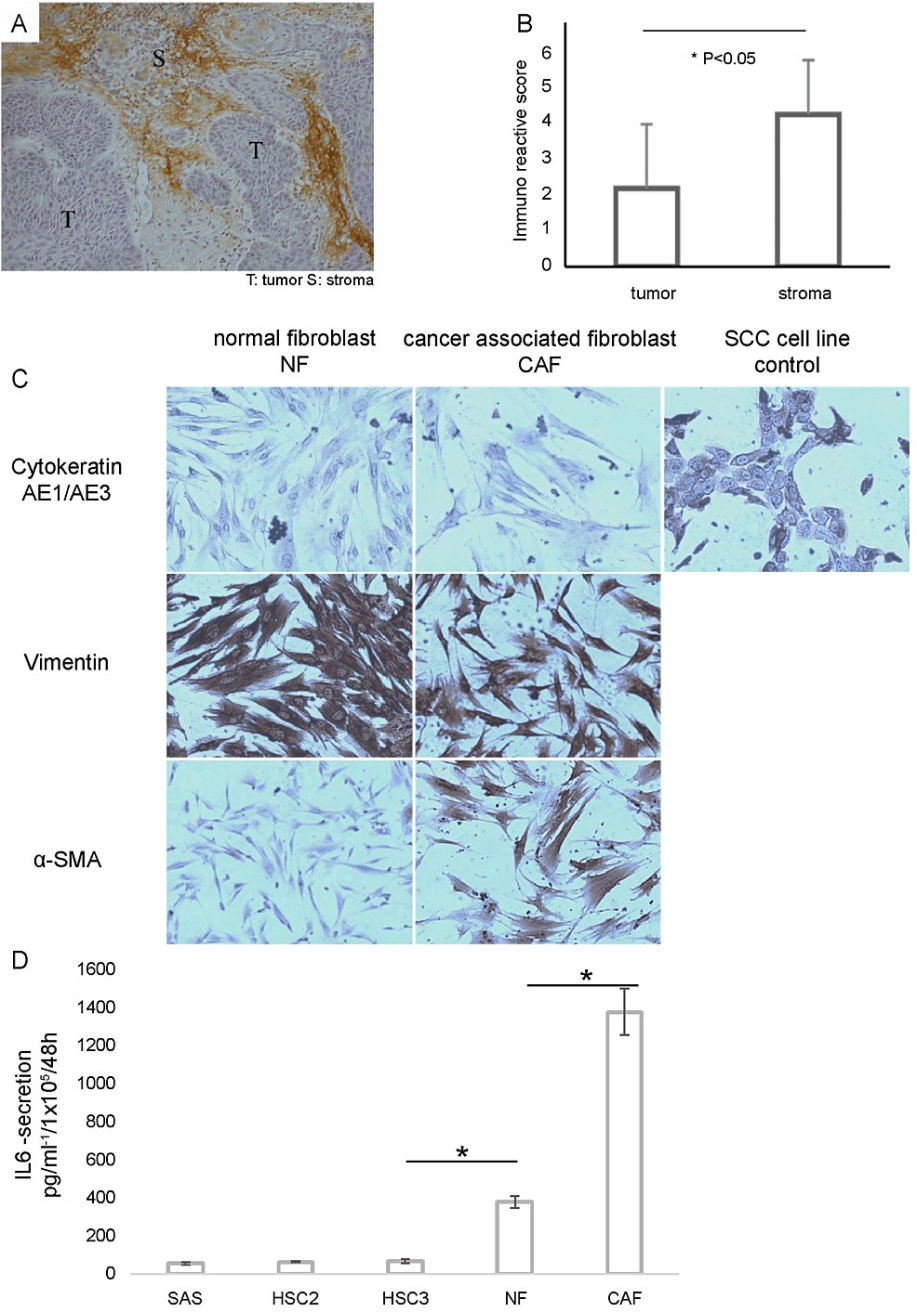
Localization of IL-6 in OSCC tissue. (A) In the OSCC tissue, IL-6 was dominantly localized in the cancer stromal compartment but was barely expressed in the tumor cells on immunohistochemistry staining. (B) Localization of IL-6 in OSCC tissue by IRS. The localization of IL-6 in cancer stroma was significantly higher than that in cancer tissue. (C) Isolation of human fibroblasts from the oral mucosa and OSCC tissue. Fibroblasts isolated from the OSCC region or normal oral mucosa were defined as cancer-associated fibroblasts (CAF) or normal fibroblasts (NF), respectively. Both CAF and NF were positive for vimentin, but only CAF was positive for αSMA. (D) Secretion of IL-6 protein in OSCC cell lines (SAS, HSC2, and HSC3) and various fibroblasts. Cancer cell lines demonstrated barely discernible IL-6 secretion, markedly contrasting with the significantly elevated secretion observed in both CAF and NF. IL-6, interleukin 6; IRS, immunoreactivity scoring system; OSCC, oral squamous cell carcinoma.

On immunohistochemical analysis to investigate the localization of IL-6 in OSCC tissues, we found that most IL-6 expression was observed in stromal cells rather than in tumor cells (Figure 2A). The immunoreactivity scoring system (IRS) showed a significant difference in IL-6 expression between tumor and stromal cells, with higher expression levels observed in stromal cells (Figure 2B; P<0.05). Fibroblasts were extracted from OSCC tissues and normal oral mucosa to confirm the expression of IL-6 and subsequently classified as cancer-associated fibroblasts (CAFs) and normal fibroblasts (NFs), respectively. Although both CAFs and NFs were cytokeratin-negative and vimentin-positive, SMA was positive only in CAFs (Figure 2C). IL-6 secretion was measured by ELISA in OSCC cell lines (SAS, HSC2, and HSC3), cancer-associated fibroblasts (CAFs), and normal fibroblasts (NFs). Although IL-6 secretion from cancer cell lines was barely detectable, that from CAF and NF was significantly higher than that from OSCC cell lines. In particular, IL-6 secretion from CAFs was three-fold higher than that from NFs (Figure 2D).

Comprehensive gene expression analysis of OSCC tissues

Our microarray-based gene expression analysis aimed to investigate the effects of IL-6 on gene expression in primary tumor tissues. The primary tumor expression profiles of nine patients with high serum IL-6 levels and nine patients with low serum IL-6 levels were compared while ensuring that clinicopathological factors, such as primary site and stage, were similar. A comparison of gene expression between the two groups was performed using a cDNA microarray. The results showed a differential gene expression profile, with 164 upregulated and 349 downregulated genes in the high IL-6 group compared with the low IL-6 group (signal noise ratio >2) (Table 2). Pathway analysis implicated genes related to oxidative reduction and lipid metabolism were over-expressed, and genes involved in cancer immunity and interferon signaling were under-expressed in the group with high serum IL-6 levels (Tables 3 and 2, respectively). The signal value of the IL-6 gene in the primary tissue was deficient in the two groups (data not shown). This may suggest that, in the group with high serum IL-6 levels, there was an increase in local metabolic activity within the tumor tissue, and the molecules involved in tumor immunity were suppressed.

**Table 2:** Top 10 variable genes in the microarray analysis.

Gene symbol	Gene title	Fold Change
FLG	filaggrin	8.610521
DAPL1	death associated protein-like 1	7.197287
KRT1	keratin 1	6.104644
SPINK5	serine peptidase inhibitor, Kazal type5	5.45473
GJB6	gap junction protein, beta 6	5.151485
A2ML1	alpha-2 macroglobulin-like 1	4.985114
RPTN	repetin	4.965783
ALOX12B	arachidonate 12-lipoxygenase, 12R type	4.888796
SCEL	sciellin	4.595318
GJB2	gap junction protein, beta 2	4.575785
IGHG1	immunoglobulin heavy constant gamma 1	-6.752317
TTN	titin	-6.811456
IGLL3	immunoglobulin lambda-like polypeptide 3	-6.964599
MGC29506	plasma cell-induced ER protein 1	-7.277525
IGH	immunoglobulin heavy locus	-7.850352
POU2AF1	POU class 2 associating factor 1	-7.869186
IGHA1	immunoglobulin heavy constant alpha 1	-9.054444
IGFG3	immunoglobulin heavy constant gamma 3	-11.2868
LOC100291682	similar to hCG1686089	-18.07968
LOC100287723	similar to the Ig kappa chain	-22.59982

**Table 3:** KEGG pathway analysis of gene expression in primary tissues.

Term	Count	%	P Value	FDR
Metabolic pathways	27	13.36364	1.34E-05	0.001065
Arachidonic acid metabolism	7	4.242424	1.90E-05	0.001065
Steroid biosynthesis	5	3.030303	2.95E-05	0.001102
Metabolism of xenobiotics by cytochrome P450	6	3.636364	5.85E-04	0.016009
Biosynthesis of antibiotics	9	5.454545	7.15E-04	0.016009
Steroid hormone biosynthesis	5	3.030303	0.001984	0.037044
Chemical carcinogenesis	5	3.030303	0.006353	0.10165
Serotonergic synapse	5	3.030303	0.019438	0.266232
Retinol metabolism	4	2.424242	0.021394	0.266232
Drug metabolism – cytochrome P450	4	2.424242	0.025071	0.28079

Association of serum IL-6 with inflammatory response and nutritional factors in patients with OSCC

**Figure 3:**
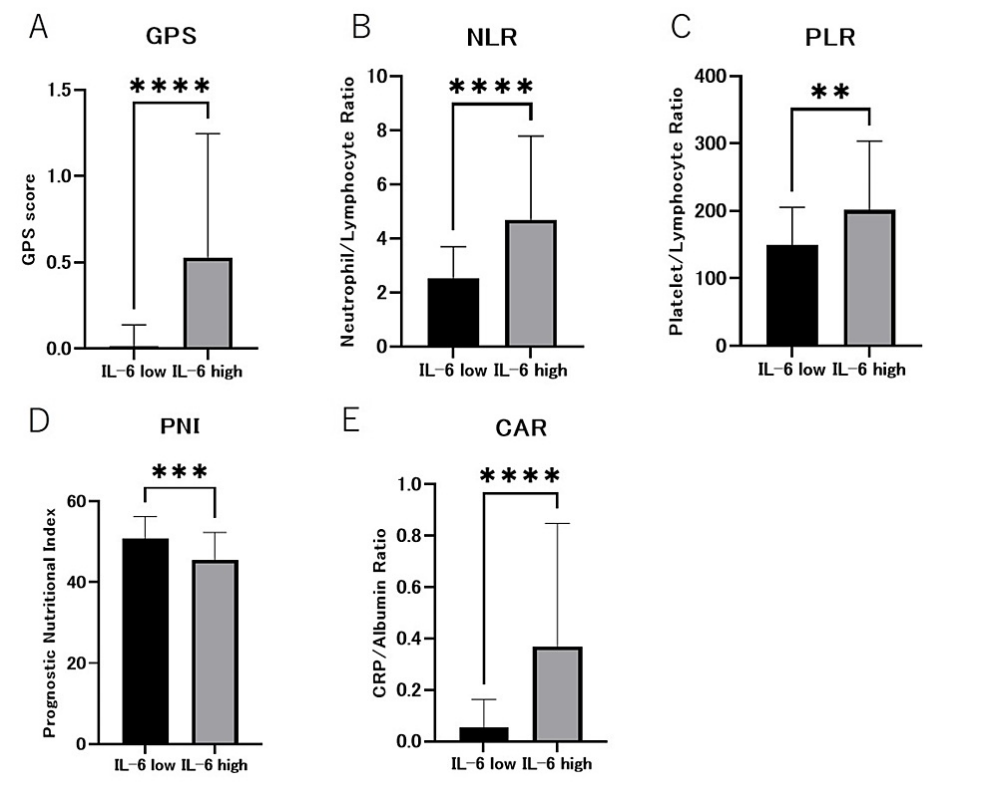
Association of serum IL-6 with inflammatory response and nutritional factors in patients with OSCC. Strong correlations were found between serum IL-6 levels and inflammatory response markers and nutritional factors. (A) Glasgow Prognostic Score (GPS), (B) Neutrophil/Lymphocyte Ratio (NLR), (C) Platelet/Lymphocyte Ratio (PLR), (D) Prognostic Nutritional Index (PNI), (E) CRP/Albumin Ratio (CAR). IL-6, interleukin 6; OSCC, oral squamous cell carcinoma. **** p<0.0001, *** p<0.001, ** p<0.01

Given the suggested association between serum IL-6 levels and metabolic abnormalities at the tumor site, we investigated the correlation between metabolic and nutritional disorders and the inflammatory response in individuals. Significant differences were observed in GPS, NLR, PLR, PNI, and CAR (Figure 3A-E). Regarding the correlation between blood tests and serum IL-6 levels, a strong positive correlation was observed between albumin (P=0.0004) and CRP levels (P=0.0001). This suggests that serum IL-6 levels may be a prognostic factor in OSCC by reflecting patient nutritional status and systemic inflammatory response.

## Discussion

Numerous biomarkers derived from clinical specimens have been reported in OSCC [4]; however, none of them are ideal for clinical application. Therefore, this study aimed to investigate the potential role of IL-6 as a prognostic marker in patients with OSCC.

Peripheral blood examination, which is capable of detecting certain factors, is a promising candidate for the evaluation of biomarkers predicting therapeutic outcomes and prognoses because of its technical ease of measurement without causing a significant burden to patients. This study showed that serum IL-6 levels in patients with OSCC correlated with prognosis. The patient group with high serum IL-6 levels had a poor prognosis, supporting previous results [5]. Several studies have suggested that IL-6 is positively correlated with advanced stages of cancer, especially T stage in patients with OSCC, indicating its association with clinical factors. Furthermore, a correlation between IL-6 and pathological factors such as the presence or absence of bone invasion, tumor invasion depth, and vascular invasion has been reported [11]. IL-6 is known to activate STAT3, which in turn upregulates the expression of cyclinD1/D2/B1 and c-Myc, and inhibits p21Cdk, thus promoting cell cycle turnover [12]. Moreover, this activation of STAT3 enhances the expression of anti-apoptotic proteins, such as Bcl-2, Bcl-XL, and Mcl-1, in cancer cells, thereby increasing resistance to cell death [13]. IL-6 has also been reported to regulate cancer cell proliferation through the epidermal growth factor (EGF) and hepatocyte growth factor (HGF) families [14]. In contrast, other reports suggest that IL-6 does not affect the growth of OSCC cell lines but influences tumor growth and progression by promoting surrounding lymphangiogenesis and angiogenesis [15,16]. There are additional reported effects of IL-6 on tumor cells, specifically regarding the migration and invasion of cancer cells and the acquisition of epithelial-mesenchymal transition (EMT) [17]. Therefore, IL-6 signaling may exogenously activate angiogenic and lymphangiogenic factors and endogenously promote the proliferation and survival of cancer cells, thereby supporting tumor growth.

In our study of IL-6 localization in OSCC tissues, we found that IL-6 expression was elevated in the stroma rather than in the tumor. To the best of our knowledge, this is a novel finding, as there have been no reports directly comparing IL-6 levels in OSCC tumors and the surrounding stroma. Nagasaki et al. reported that stromal fibroblasts in colon cancer produced significant amounts of IL-6. Cancer cells enhance IL-6 production by fibroblasts, and IL-6 increases vascular endothelial growth factor (VEGF) production, promoting angiogenesis [18]. IL-6 secreted by CAFs has been reported to promote epithelial-mesenchymal transition and metastasis of gastric cancer via the JAK2/STAT3 signaling pathway [19]. Furthermore, IL-6 from CAFs has also been linked to the induction of epithelial-mesenchymal transition, proliferation, migration, and invasion of bladder cancer cells [20]. Recently, the notion that CAFs comprise a heterogeneous group of cells has been proposed. Advances in technology, such as single-cell RNA sequencing, have allowed for a detailed understanding of heterogeneity within the CAF population. Therefore, the investigation of fibroblast diversity and its therapeutic potential is a rapidly progressing subject for future research.

To date, the main reports on IL-6 in OSCC have focused on its prognostic value in serum levels, functional analysis of cell lines, and systemic effects, such as involvement in cachexia. In this study, we performed a microarray analysis of tumor tissues with high and low serum IL-6 levels. The signal values of IL-6 in tumor tissues were low, regardless of serum IL-6 levels. The gene ontology (GO) analysis showed that activation of metabolic pathways, such as arachidonic acid metabolism and steroid biosynthesis, were observed in the group with high serum IL-6 levels. Recently, cancer metabolism, specifically the energy metabolism of cancer cells, has gained significant attention in cancer biology. It is now considered a fundamental area of research along with signal transduction and transcription [21]. Several metabolic pathways, including glycolysis and lipid metabolism, have been identified to be associated with OSCC [22], and there have been reports on their potential utility as prognostic factors [23]. COXs and lipoxygenases produce bioactive lipids from arachidonic acid, which are involved in tumorigenesis and play an important role in prostate cancer [24]. Further investigations are warranted based on the correlation between serum IL-6 levels and abnormalities in these metabolic pathways, indicating their potential as novel therapeutic targets in cancer. However, it has been reported that prospective studies continuously assessing serum IL-6 levels in OSCC patients are important for further understanding its potential as a biomarker and its association with disease progression [25].

Although this study has demonstrated that patients with elevated levels of IL-6 in their serum also displayed augmented local metabolic activity in cancerous tissues, the systemic repercussions of this finding remain ambiguous. Additionally, the impact of cancer on resting energy expenditure and other parameters has yet to be determined. This study also found significant differences in the PLR, NLR, CAR, PNI, and GPS indices of inflammation and nutritional status, depending on serum IL-6 levels. These findings suggest that patients with OSCC exhibit elevated systemic inflammatory responses, hyponutrition, and heightened local metabolic activity, which may be the underlying cause of cachexia and cancer-induced weight loss. In experimental animal models, administration of monoclonal antibodies against IL-6 or an IL-6 receptor antagonist significantly inhibited the progression of cancer cachexia in tumor-bearing mice [26]. This demonstrates that IL-6 is a causal factor in cancer cachexia, regardless of tumor growth.

Recently, the importance of nutritional therapy in cancer treatment has garnered significant attention. Therefore, all individuals diagnosed with cancer require nutritional assessments at diagnosis, and nutritional interventions should be implemented before their general health status declines [27]. Nutritional interventions for head, neck, and gastrointestinal cancers have been shown to reduce weight and quality of life. Animal models of cachexia have been reported for pancreatic cancer. The details of the mechanisms involving IL-6 are being investigated, suggesting that they may be a direct target for treatment [28]. Prolonged patient survival and improved quality of life have been reported following the administration of anti-IL-6 receptor antibodies for cachexia in patients with lung cancer [29]. Furthermore, various cytokine-inhibitory agents have been examined for their ability to disable cachexia-related cytokines. Eicosapentaenoic acid, an α-3-ω fatty acid present in fish, was found to suppress IL-6 production in patients with cachexia, and its efficacy has been evaluated in randomized trials [30]. Serum IL-6 levels in patients with OSCC may be valuable markers for identifying high-risk patients and predicting treatment resistance and prognosis due to metabolic and nutritional disorders. Therefore, it is essential to consider treatment options targeting IL-6 or aggressive nutritional therapy in patients with OSCC in the future.

OSCC is still considered a rare cancer in Japan, resulting in a small number of cases in this study. It is necessary to increase the sample size and conduct prospective trials, including regular measurements, in the future.

## Conclusions

In this study, it was suggested that serum IL-6 levels in patients with OSCC might serve as a valuable marker for predicting prognosis. The basis for this includes the strong positive correlation with CRP and strong negative correlation with Alb among blood biochemistry tests. Furthermore, comprehensive gene expression analysis in cancer sites revealed an upregulation of metabolism-related genes in the group with high serum IL-6 levels. Localization of IL-6 in cancer tissue was predominantly found in the stroma, especially in cancer-associated fibroblasts, rather than in cancer cells. These results could serve as a guideline for distinguishing potentially highly malignant tumors in future OSCC treatments. 
